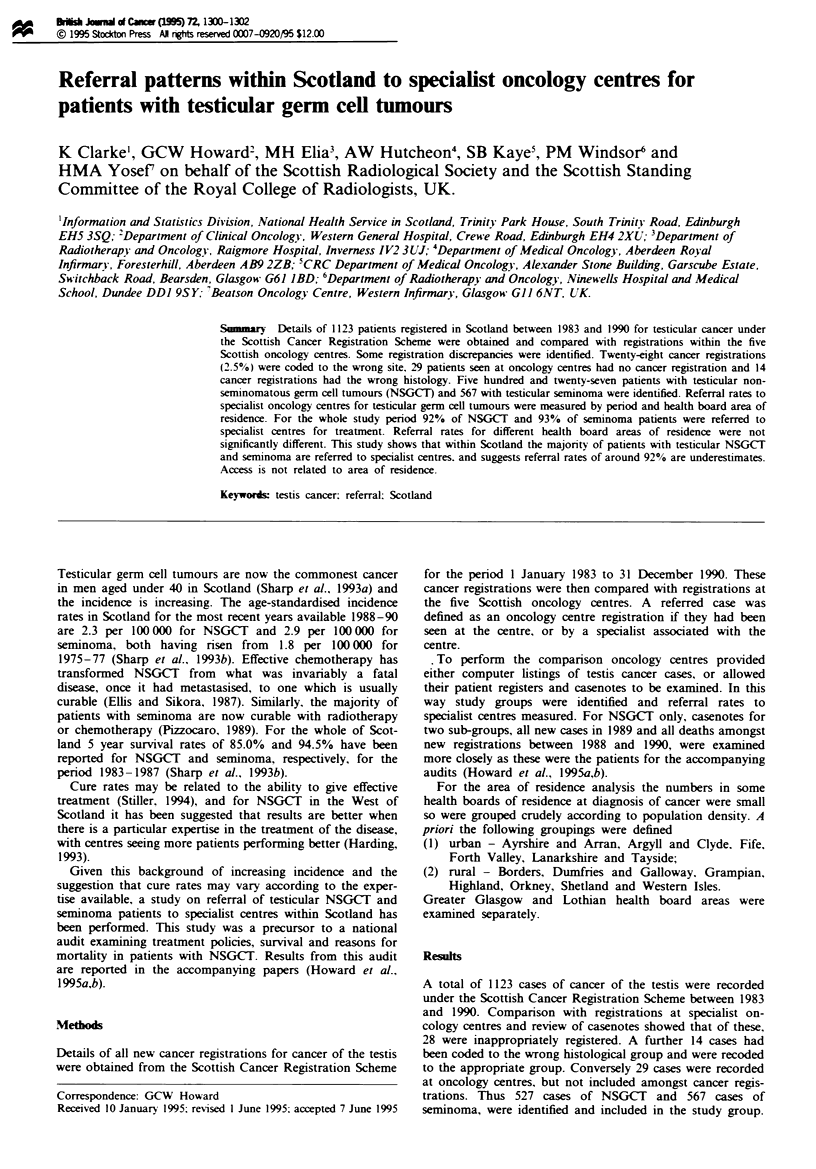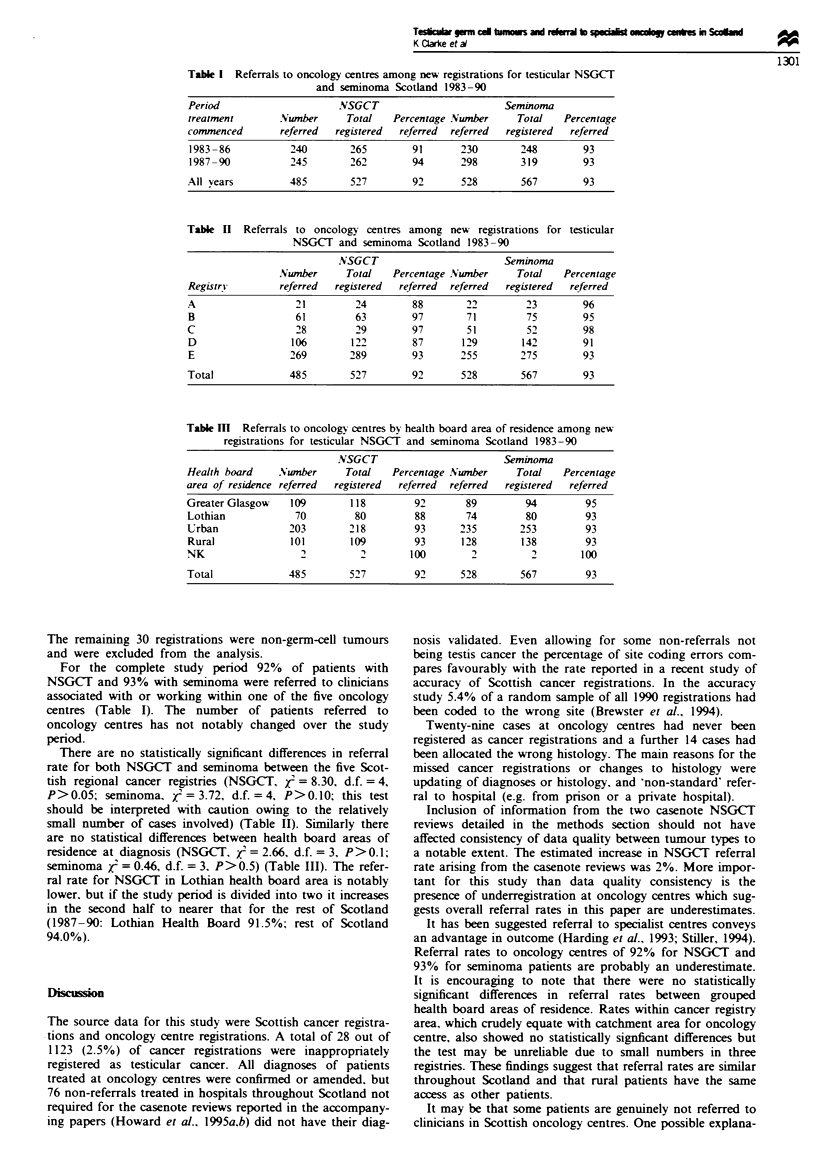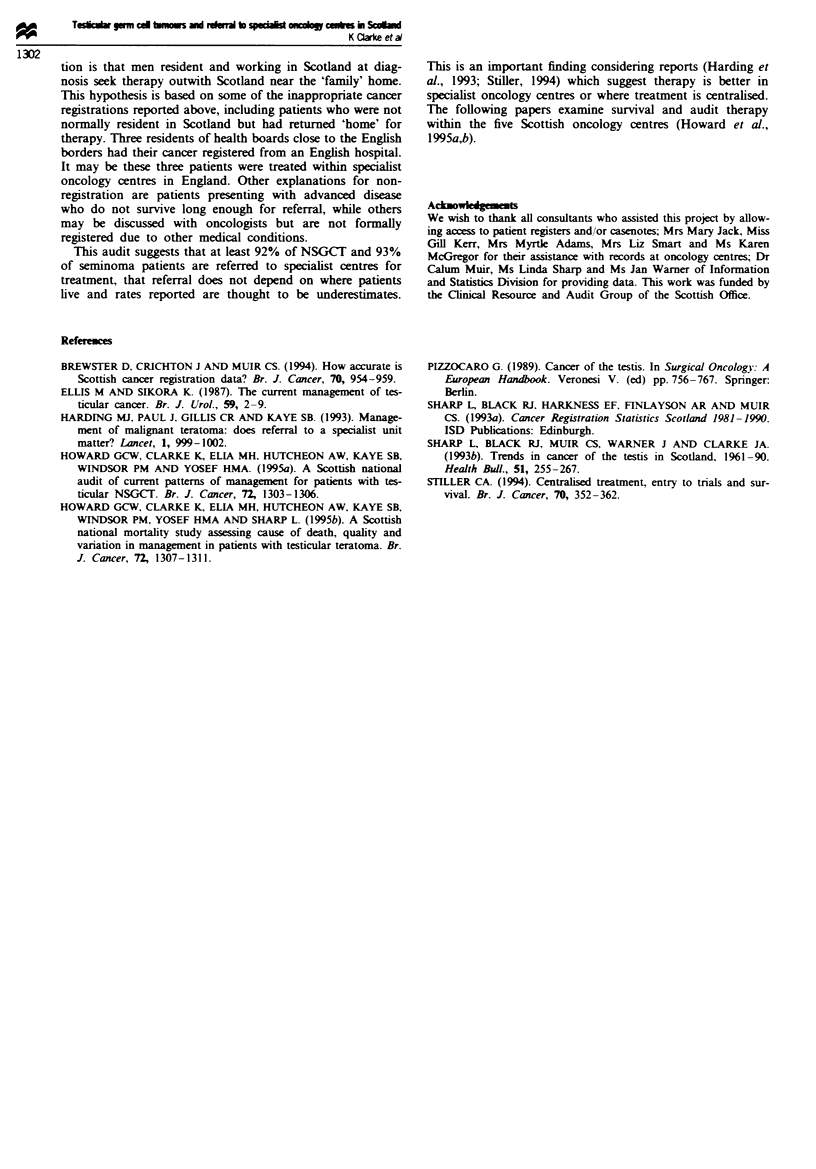# Referral patterns within Scotland to specialist oncology centres for patients with testicular germ cell tumours. The Scottish Radiological Society and the Scottish Standing Committee of the Royal College of Radiologists.

**DOI:** 10.1038/bjc.1995.504

**Published:** 1995-11

**Authors:** K. Clarke, G. C. Howard, M. H. Elia, A. W. Hutcheon, S. B. Kaye, P. M. Windsor, H. M. Yosef

**Affiliations:** Information and Statistics Division, National Health Service in Scotland, Edinburgh, UK.

## Abstract

Details of 1123 patients registered in Scotland between 1983 and 1990 for testicular cancer under the Scottish Cancer Registration Scheme were obtained and compared with registrations within the five Scottish oncology centres. Some registration discrepancies were identified. Twenty-eight cancer registrations (2.5%) were coded to the wrong site, 29 patients seen at oncology centres had no cancer registration and 14 cancer registrations had the wrong histology. Five hundred and twenty-seven patients with testicular non-seminomatous germ cell tumours (NSGCT) and 567 with testicular seminoma were identified. Referral rates to specialist oncology centres for testicular germ cell tumours were measured by period and health board area of residence. For the whole study period 92% of NSGCT and 93% of seminoma patients were referred to specialist centres for treatment. Referral rates for different health board areas of residence were not significantly different. This study shows that within Scotland the majority of patients with testicular NSGCT and seminoma are referred to specialist centres, and suggests referral rates of around 92% are underestimates. Access is not related to area of residence.


© 1995 Stockton Press All rights reserved 0007-0920/95 $12.00

### Referral patterns within Scotland to specialist oncology centres for patients with testicular germ cell tumours

K Clarke[1], GCW Howard[2], MH Elia[3], AW Hutcheon[4], SB Kaye[5], PM Windsor[6] and HMA Yosef[7] on behalf of the Scottish Radiological Society and the Scottish Standing Committee of the Royal College of Radiologists, UK.

[1]*Information and Statistics Division, National Health Service in Scotland, Trinity Park House, South Trinity Road, Edinburgh EH5 3SQ;* [2]*Department of Clinical Oncology, Western General Hospital, Crewe Road, Edinburgh EH4 2XU;* [3]*Department of Radiotherapy and Oncology, Raigmore Hospital, Inverness IV2 3UJ;* [4]*Department of Medical Oncology, Aberdeen Royal Infirmary, Foresterhill, Aberdeen AB9 2ZB;* [5]*CRC Department of Medical Oncology, Alexander Stone Building, Garscube Estate, Switchback Road, Bearsden, Glasgow G61 1BD;* [6]*Department of Radiotherapy and Oncology, Ninewells Hospital and Medical School, Dundee DD1 9SY;* [7]*Beatson Oncology Centre, Western Infirmary, Glasgow G11 6NT, UK.*

**Summary** Details of 1123 patients registered in Scotland between 1983 and 1990 for testicular cancer under the Scottish Cancer Registration Scheme were obtained and compared with registrations within the five Scottish oncology centres. Some registration discrepancies were identified. Twenty-eight cancer registrations (2.5%) were coded to the wrong site, 29 patients seen at oncology centres had no cancer registration and 14 cancer registrations had the wrong histology. Five hundred and twenty-seven patients with testicular non-seminomatous germ cell tumours (NSGCT) and 567 with testicular seminoma were identified. Referral rates to specialist oncology centres for testicular germ cell tumours were measured by period and health board area of residence. For the whole study period 92% of NSGCT and 93% of seminoma patients were referred to specialist centres for treatment. Referral rates for different health board areas of residence were not significantly different. This study shows that within Scotland the majority of patients with testicular NSGCT and seminoma are referred to specialist centres, and suggests referral rates of around 92% are underestimates. Access is not related to area of residence.

**Keywords:** testis cancer; referral; Scotland

Testicular germ cell tumours are now the commonest cancer in men aged under 40 in Scotland (Sharp *et al.*, 1993*a*) and the incidence is increasing. The age-standardised incidence rates in Scotland for the most recent years available 1988-90 are 2.3 per 100 000 for NSGCT and 2.9 per 100 000 for seminoma, both having risen from 1.8 per 100 000 for 1975-77 (Sharp *et al.*, 1993*b*). Effective chemotherapy has transformed NSGCT from what was invariably a fatal disease, once it had metastasised, to one which is usually curable (Ellis and Sikora, 1987). Similarly, the majority of patients with seminoma are now curable with radiotherapy or chemotherapy (Pizzocaro, 1989). For the whole of Scotland 5 year survival rates of 85.0% and 94.5% have been reported for NSGCT and seminoma, respectively, for the period 1983-1987 (Sharp *et al.*, 1993*b*).

Cure rates may be related to the ability to give effective treatment (Stiller, 1994), and for NSGCT in the West of Scotland it has been suggested that results are better when there is a particular expertise in the treatment of the disease, with centres seeing more patients performing better (Harding, 1993).

Given this background of increasing incidence and the suggestion that cure rates may vary according to the expertise available, a study on referral of testicular NSGCT and seminoma patients to specialist centres within Scotland has been performed. This study was a precursor to a national audit examining treatment policies, survival and reasons for mortality in patients with NSGCT. Results from this audit are reported in the accompanying papers (Howard *et al.*, 1995*a*,*b*).

### Methods

Details of all new cancer registrations for cancer of the testis were obtained from the Scottish Cancer Registration Scheme for the period 1 January 1983 to 31 December 1990. These cancer registrations were then compared with registrations at the five Scottish oncology centres. A referred case was defined as an oncology centre registration if they had been seen at the centre, or by a specialist associated with the centre.

To perform the comparison oncology centres provided either computer listings of testis cancer cases, or allowed their patient registers and casenotes to be examined. In this way study groups were identified and referral rates to specialist centres measured. For NSGCT only, casenotes for two sub-groups, all new cases in 1989 and all deaths amongst new registrations between 1988 and 1990, were examined more closely as these were the patients for the accompanying audits (Howard *et al.*, 1995*a*,*b*).

For the area of residence analysis the numbers in some health boards of residence at diagnosis of cancer were small so were grouped crudely according to population density. *A priori* the following groupings were defined

(1) urban - Ayrshire and Arran, Argyll and Clyde, Fife, Forth Valley, Lanarkshire and Tayside;
(2) rural - Borders, Dumfries and Galloway, Grampian, Highland, Orkney, Shetland and Western Isles.

Greater Glasgow and Lothian health board areas were examined separately.

### Results

A total of 1123 cases of cancer of the testis were recorded under the Scottish Cancer Registration Scheme between 1983 and 1990. Comparison with registrations at specialist oncology centres and review of casenotes showed that of these, 28 were inappropriately registered. A further 14 cases had been coded to the wrong histological group and were recoded to the appropriate group. Conversely 29 cases were recorded at oncology centres, but not included amongst cancer registrations. Thus 527 cases of NSGCT and 567 cases of seminoma, were identified and included in the study group.

Correspondence: GCW Howard
Received 10 January 1995; revised 1 June 1995; accepted 7 June 1995

Table I Referrals to oncology centres among new registrations for testicular NSGCT and seminoma Scotland 1983–90

| *Period treatment commenced* | *NSGCT* *Number referred* | *Total registered* | *Percentage referred* | *Seminoma* *Number referred* | *Total registered* | *Percentage referred* |
|---|---|---|---|---|---|---|
| 1983–86 | 240 | 265 | 91 | 230 | 248 | 93 |
| 1987–90 | 245 | 262 | 94 | 298 | 319 | 93 |
| All years | 485 | 527 | 92 | 528 | 567 | 93 |

Table II Referrals to oncology centres among new registrations for testicular NSGCT and seminoma Scotland 1983–90

| *Registry* | *NSGCT* *Number referred* | *Total registered* | *Percentage referred* | *Seminoma* *Number referred* | *Total registered* | *Percentage referred* |
|---|---|---|---|---|---|---|
| A | 21 | 24 | 88 | 22 | 23 | 96 |
| B | 61 | 63 | 97 | 71 | 75 | 95 |
| C | 28 | 29 | 97 | 51 | 52 | 98 |
| D | 106 | 122 | 87 | 129 | 142 | 91 |
| E | 269 | 289 | 93 | 255 | 275 | 93 |
| Total | 485 | 527 | 92 | 528 | 567 | 93 |

Table III Referrals to oncology centres by health board area of residence among new registrations for testicular NSGCT and seminoma Scotland 1983–90

| *Health board area of residence* | *NSGCT* *Number referred* | *Total registered* | *Percentage referred* | *Seminoma* *Number referred* | *Total registered* | *Percentage referred* |
|---|---|---|---|---|---|---|
| Greater Glasgow | 109 | 118 | 92 | 89 | 94 | 95 |
| Lothian | 70 | 80 | 88 | 74 | 80 | 93 |
| Urban | 203 | 218 | 93 | 235 | 253 | 93 |
| Rural | 101 | 109 | 93 | 128 | 138 | 93 |
| NK | 2 | 2 | 100 | 2 | 2 | 100 |
| Total | 485 | 527 | 92 | 528 | 567 | 93 |

The remaining 30 registrations were non-germ-cell tumours and were excluded from the analysis.

For the complete study period 92% of patients with NSGCT and 93% with seminoma were referred to clinicians associated with or working within one of the five oncology centres (Table I). The number of patients referred to oncology centres has not notably changed over the study period.

There are no statistically significant differences in referral rate for both NSGCT and seminoma between the five Scottish regional cancer registries (NSGCT, $\chi^2 = 8.30$, d.f. = 4, $P > 0.05$; seminoma, $\chi^2 = 3.72$, d.f. = 4, $P > 0.10$; this test should be interpreted with caution owing to the relatively small number of cases involved) (Table II). Similarly there are no statistical differences between health board areas of residence at diagnosis (NSGCT, $\chi^2 = 2.66$, d.f. = 3, $P > 0.1$; seminoma $\chi^2 = 0.46$, d.f. = 3, $P > 0.5$) (Table III). The referral rate for NSGCT in Lothian health board area is notably lower, but if the study period is divided into two it increases in the second half to nearer that for the rest of Scotland (1987–90: Lothian Health Board 91.5%; rest of Scotland 94.0%).

### Discussion

The source data for this study were Scottish cancer registrations and oncology centre registrations. A total of 28 out of 1123 (2.5%) of cancer registrations were inappropriately registered as testicular cancer. All diagnoses of patients treated at oncology centres were confirmed or amended, but 76 non-referrals treated in hospitals throughout Scotland not required for the casenote reviews reported in the accompanying papers (Howard *et al.*, 1995*a*,*b*) did not have their diagnosis validated. Even allowing for some non-referrals not being testis cancer the percentage of site coding errors compares favourably with the rate reported in a recent study of accuracy of Scottish cancer registrations. In the accuracy study 5.4% of a random sample of all 1990 registrations had been coded to the wrong site (Brewster *et al.*, 1994).

Twenty-nine cases at oncology centres had never been registered as cancer registrations and a further 14 cases had been allocated the wrong histology. The main reasons for the missed cancer registrations or changes to histology were updating of diagnoses or histology, and 'non-standard' referral to hospital (e.g. from prison or a private hospital).

Inclusion of information from the two casenote NSGCT reviews detailed in the methods section should not have affected consistency of data quality between tumour types to a notable extent. The estimated increase in NSGCT referral rate arising from the casenote reviews was 2%. More important for this study than data quality consistency is the presence of underregistration at oncology centres which suggests overall referral rates in this paper are underestimates.

It has been suggested referral to specialist centres conveys an advantage in outcome (Harding *et al.*, 1993; Stiller, 1994). Referral rates to oncology centres of 92% for NSGCT and 93% for seminoma patients are probably an underestimate. It is encouraging to note that there were no statistically significant differences in referral rates between grouped health board areas of residence. Rates within cancer registry area, which crudely equate with catchment area for oncology centre, also showed no statistically signficant differences but the test may be unreliable due to small numbers in three registries. These findings suggest that referral rates are similar throughout Scotland and that rural patients have the same access as other patients.

It may be that some patients are genuinely not referred to clinicians in Scottish oncology centres. One possible explanation is that men resident and working in Scotland at diagnosis seek therapy outwith Scotland near the 'family' home. This hypothesis is based on some of the inappropriate cancer registrations reported above, including patients who were not normally resident in Scotland but had returned 'home' for therapy. Three residents of health boards close to the English borders had their cancer registered from an English hospital. It may be these three patients were treated within specialist oncology centres in England. Other explanations for non-registration are patients presenting with advanced disease who do not survive long enough for referral, while others may be discussed with oncologists but are not formally registered due to other medical conditions.

This audit suggests that at least 92% of NSGCT and 93% of seminoma patients are referred to specialist centres for treatment, that referral does not depend on where patients live and rates reported are thought to be underestimates. This is an important finding considering reports (Harding *et al*., 1993; Stiller, 1994) which suggest therapy is better in specialist oncology centres or where treatment is centralised. The following papers examine survival and audit therapy within the five Scottish oncology centres (Howard *et al*., 1995*a*,*b*).

**Acknowledgements**
We wish to thank all consultants who assisted this project by allowing access to patient registers and/or casenotes; Mrs Mary Jack, Miss Gill Kerr, Mrs Myrtle Adams, Mrs Liz Smart and Ms Karen McGregor for their assistance with records at oncology centres; Dr Calum Muir, Ms Linda Sharp and Ms Jan Warner of Information and Statistics Division for providing data. This work was funded by the Clinical Resource and Audit Group of the Scottish Office.

**References**

BREWSTER D, CRICHTON J AND MUIR CS. (1994). How accurate is Scottish cancer registration data? *Br. J. Cancer*, **70**, 954-959.

ELLIS M AND SIKORA K. (1987). The current management of testicular cancer. *Br. J. Urol*., **59**, 2-9.

HARDING MJ, PAUL J, GILLIS CR AND KAYE SB. (1993). Management of malignant teratoma: does referral to a specialist unit matter? *Lancet*, **1**, 999-1002.

HOWARD GCW, CLARKE K, ELIA MH, HUTCHEON AW, KAYE SB, WINDSOR PM AND YOSEF HMA. (1995*a*). A Scottish national audit of current patterns of management for patients with testicular NSGCT. *Br. J. Cancer*, **72**, 1303-1306.

HOWARD GCW, CLARKE K, ELIA MH, HUTCHEON AW, KAYE SB, WINDSOR PM, YOSEF HMA AND SHARP L. (1995*b*). A Scottish national mortality study assessing cause of death, quality and variation in management in patients with testicular teratoma. *Br. J. Cancer*, **72**, 1307-1311.

PIZZOCARO G. (1989). Cancer of the testis. In *Surgical Oncology: A European Handbook*. Veronesi V. (ed) pp. 756-767. Springer: Berlin.

SHARP L, BLACK RJ, HARKNESS EF, FINLAYSON AR AND MUIR CS. (1993*a*). *Cancer Registration Statistics Scotland 1981-1990*. ISD Publications: Edinburgh.

SHARP L, BLACK RJ, MUIR CS, WARNER J AND CLARKE JA. (1993*b*). Trends in cancer of the testis in Scotland, 1961-90. *Health Bull*., **51**, 255-267.

STILLER CA. (1994). Centralised treatment, entry to trials and survival. *Br. J. Cancer*, **70**, 352-362.